# Patient Perspectives on Blended Internet-Based and Face-to-Face Cognitive Behavioral Therapy for Alcohol Use Disorder: Qualitative Study

**DOI:** 10.2196/47083

**Published:** 2024-10-23

**Authors:** Kristine Tarp, Regina Christiansen, Randi Bilberg, Simone Borkner, Caroline Dalsgaard, Marie Paldam Folker, Anette Søgaard Nielsen

**Affiliations:** 1 Research Unit for Digital Psychiatry Department of Clinical Research University of Southern Denmark Odense Denmark; 2 Centre for Digital Psychiatry Mental Health Services Region of Southern Denmark Odense Denmark; 3 The National Research Centre for the Working Environment Copenhagen Denmark; 4 Unit of Clinical Alcohol Research Department of Clinical Research University of Southern Denmark Odense Denmark; 5 Psychiatric University Hospital University Function Region of Southern Denmark Odense Denmark; 6 Department for finance and planning Lillebaelt Hospital University Hospital of Southern Denmark Vejle Denmark; 7 Brain Research, Inter-Disciplinary Guided Excellence Department of Clinical Research University of Southern Denmark Odense Denmark; 8 OPEN Open Patient data Explorative Network Odense University Hospital Odense Denmark

**Keywords:** internet-based, alcohol use disorder treatment, user perspective, qualitative, blended treatment, blended learning, cognitive behavioral therapy, alcoholism, alcohol use disorder, treatment, barriers, patient perspectives, rehabilitation

## Abstract

**Background:**

Harmful alcohol consumption has been identified as a major contributor to disease, mortality, and social harm, accounting for 5.3% of worldwide deaths annually. In Denmark, an estimated 150,000 people suffer from alcohol use disorder (AUD), but a low proportion seek treatment due to person- and treatment-related barriers. Internet-based cognitive behavioral therapy (iCBT) has shown positive effects on the treatment gap, with patients reporting benefits such as increased knowledge and flexibility. However, there is a lack of research on blended cognitive behavioral therapy (bCBT), which combines face-to-face CBT (FtF CBT) and iCBT for AUD.

**Objective:**

This study aims to investigate user experiences of bCBT. More specifically, it seeks to explore the advantages and disadvantages that users have experienced with bCBT for AUD, as well as their motivations for choosing this treatment format.

**Methods:**

A total of 30 patients who had participated in the Blend-A (Blending Internet Treatment into Conventional Face-to-Face Treatment for AUD) study and received the intervention were contacted and offered the opportunity to participate in semistructured individual telephone interviews. Of these, 12 patients consented to participate. Furthermore, an additional participant was approached at a municipal clinic and agreed to engage in an individual FtF interview. Thus, the final sample consisted of 13 patients. The interviews explored their background, experiences with digital technology, motivations for choosing internet-based treatment, and experiences with the program during AUD treatment. The interviews were audio-recorded and transcribed in full length and analyzed using thematic analysis. All data were anonymized and securely stored.

**Results:**

We found that users experienced several advantages of iCBT over a larger part of the treatment course, including increased anonymity and privacy. Most importantly, it offered flexibility, allowing patients to focus on their rehabilitation process at their own pace. Patients appreciated the availability of written text in the online program, finding it helpful for gaining knowledge and understanding of AUD and its impact on the individual with the condition. They emphasized how the assignments helped them fully engage in treatment by first acknowledging their problem with alcohol and then dedicating time to self-reflection before FtF sessions, allowing for more in-depth discussions with the therapist. They also appreciated the reminders, which motivated them to complete their assignments.

**Conclusions:**

Overall, patients perceived more benefits than disadvantages in using bCBT. Essentially, bCBT offers a form of assisted autonomy that cannot be fully achieved through iCBT or FtF CBT alone. It is only through their combination that patients can fully appreciate the benefits of the treatment, as they have time for self-reflection, with guidance from the therapist between FtF CBT sessions.

**Trial Registration:**

ClinicalTrials.gov NCT04535258; https://clinicaltrials.gov/ct2/show/NCT04535258

## Introduction

### Background

Unhealthy alcohol consumption represents a significant public health concern globally, contributing to increased risks of disease, mortality, and social harm [1]. It is estimated that 100 million people worldwide, or 1.3% of the total adult population, suffer from an alcohol use disorder (AUD) [2]. In Denmark, approximately 150,000 adults are estimated to be dependent drinkers, accounting for about 3% of the total adult population [3]. Despite the availability of free and easily accessible treatment for AUD in Denmark, the proportion of Danes seeking treatment remains low. This phenomenon is believed to stem from various factors, including a lack of awareness about available treatment options, insufficient perceived need for intervention, and concerns regarding the social stigma associated with seeking help for AUDs [4].

To address this treatment gap, internet-based modalities such as internet-based cognitive behavioral therapy (iCBT) have emerged as promising alternatives, effective in reducing alcohol consumption [1]. Patients who have participated in iCBT for mental health issues, including AUD, have reported that the treatment was helpful. For example, in a user experience study on internet-based treatment for problematic alcohol use, Ekström and Johansson [5] found that various components of iCBT, such as therapist support and discussion forums, were perceived as helpful and useful by patients, particularly when they could relate to the content, which in turn facilitated reflection on their alcohol consumption. In a qualitative study on user motivations and experiences with engagement in an internet intervention for alcohol reduction, Black et al [1] found that evidence-based behavior change techniques, such as social support, normative strategies, goal setting, and self-monitoring, were acceptable to patients. Similarly, in a qualitative study on experiences with iCBT for depression and anxiety, Lindegaard et al [6] found that patients experienced varying levels of success in applying the new information they had learned from treatment to their everyday lives.

Furthermore, patients reported that iCBT offered greater flexibility and accessibility compared with face-to-face (FtF) treatment, making it easier to adapt to changing routines for self-help tools and monitoring. For example, in the user experience study by Ekström and Johansson [5], availability was identified as an important factor for patients when choosing iCBT. In a review of women’s expectations and experiences regarding eHealth treatment, Verhoeks et al [7] found that eHealth lowered the threshold for women seeking health care, as barriers to eHealth treatment, such as time constraints, were less significant compared with those for FtF treatment.

Moreover, iCBT may provide opportunities for discretion and anonymity. For instance, in a qualitative study on individuals seeking online help to reduce their drinking, Khadjesari et al [8] found that patients viewed the privacy of the internet as an important factor when searching for assistance, as online help allowed them to avoid the stigma and embarrassment often associated with seeking help in person. Similarly, Ekström and Johansson [5] identified anonymity as a crucial factor for patients when choosing internet-based support, noting that feelings of shame were among the reasons patients decided to reduce their drinking. Verhoeks et al [7] reported that barriers to eHealth treatment, such as feelings of shame, were less significant compared with those associated with FtF treatment. Results from Lindegaard et al [6] also indicated that iCBT could, to some extent, mitigate mental health stigma.

In addition, iCBT addressing mental health issues may be particularly useful for individuals who do not perceive their problems as serious enough for traditional treatment, as they can skip irrelevant modules in iCBT. For instance, the study by Black et al [1] found it possible to incorporate evidence-based behavior change techniques, such as social support, normative strategies, goal setting, and self-monitoring, into iCBT to engage patients who might otherwise delay or refuse help-seeking. Results from Lindegaard et al [6] also indicate that participation in iCBT can serve as a precursor to seeking other forms of treatment.

However, iCBT also presents challenges, particularly in ensuring patient adherence to the program. Verhoeks et al [7] found that reduced feelings of obligation and a lack of motivation were the greatest challenges women faced in completing eHealth treatment. The women expressed a desire for more support during treatment, preferably through blended care. To address this, a blended approach that combines FtF CBT with iCBT—known as blended cognitive behavioral therapy (bCBT)—has been proposed [9]. This blended approach aims to capitalize on the advantages of both formats, potentially enhancing treatment accessibility, reducing stigma, and providing a more tailored treatment experience. While FtF CBT offers the benefits of personal interaction, immediate feedback, and tailored adjustments, iCBT allows for deeper reflection and exploration [10]. Combining these formats into bCBT may therefore present a meaningful approach.

Despite the theoretical benefits associated with bCBT, the literature currently lacks empirical investigations into its effectiveness in the treatment of AUD. To the authors’ knowledge, this specific area of research remains unexplored. This study aims to address this gap by providing qualitative insights from the Blend-A Study (*Blending Internet Treatment into Conventional Face-to-Face Treatment for AUD*), which investigates the experiences of patients engaged in bCBT for AUD.

### Aim

This study aims to uncover patients’ perceptions and experiences related to the use of bCBT. Based on interviews with patients participating in the Blend-A Study, we present the findings in 2 categories: (1) patient experiences regarding bCBT as a treatment form and (2) patient experiences with the practical use of the platform.

## Methods

### Design

This study used a qualitative design based on semistructured interviews with patients participating in bCBT for AUD. We followed the COREQ (Consolidated Criteria for Reporting Qualitative Research) [11] checklist for reporting our findings.

### Setting

In Denmark, AUD treatment is provided through municipalities at no cost to patients. The treatment primarily consists of evidence-based methods such as CBT and motivational interviewing [12]. Conventional AUD treatment is often delivered through individual FtF sessions or group therapy, with the duration of treatment varying between 4 and 6 months, depending on the patient’s goals and challenges. The staff at treatment centers typically includes nurses, psychologists, pedagogues, and social workers who specialize in AUD treatment and are referred to as therapists.

The implementation of the Blend-A Study commenced in June 2020 and continued until the end of December 2022. A total of 18 Danish municipal treatment institutions participated; however, 4 dropped out during the study due to resource challenges, which hindered their ability to dedicate time and focus on project meetings, patient recruitment, and the implementation of the blended format.

The Blend-A treatment program protocol originated from the Netherlands [13] and was translated and adjusted to better align with the Danish language and culture [14]. The Blend-A intervention is a form of bCBT, grounded in CBT and motivational interviewing. This blended format allows for the integration and substitution of FtF sessions within a treatment course with internet-based modules, which are available on a web-based treatment platform. The frequency of FtF sessions and the balance between FtF sessions and the use of internet-based modules were agreed upon by the patient and therapist. Typically, patients had a few FtF sessions before engaging with the internet-based modules. Patients often met FtF every 2-4 weeks, working on the modules on the platform in between. However, some patients utilized the platform almost exclusively, attending only 1 or 2 initial FtF sessions and possibly a later follow-up session, if any.

Based on experiences from the pilot phase, the internet-based platform was hosted by the Dutch company Minddistrict. It was accessible online via any web browser and offered a flexible format, allowing patients to access the platform according to their individual daily routines. Patients could also access the platform anonymously, if preferred. The platform included 21 online sessions, maintenance modules, and diaries, which the therapist could gradually add to the patient’s individual account based on their treatment goals and challenges. The first 12 sessions were considered core sessions and were offered to all patients engaged in bCBT, while the subsequent sessions were optional and tailored to the patient’s needs. Each session followed a fixed structure, starting with information about the background of the specific session, followed by multiple exercises and assignments. The online sessions included textboxes and videos. In most sessions, patients received online feedback from their therapist on the assignments they completed, which therapists aimed to provide once a week at a set time. After receiving feedback on their assignments, the next session would be made available to the patient by the therapist. Upon completing treatment, patients continued to have access to the online treatment platform, allowing them to re-read information and review previously completed exercises. An overview of the platform sessions can be seen in Table 1.

**Table 1:** Platform sessions.

Session	Content	Activity
Session 1	Welcome to the Blend-A^a^ online treatment	N/A^b^
Session 2	Support from your social network	N/A
Session 3	Explanation of treatment course	N/A
Session 4	Preparation to change	Feedback moment
Session 5	Goals and techniques for self-control	Feedback moment
Session 6	List of alcohol use risk situations	N/A
Session 7	Alcohol use function analysis	Feedback moment
Session 8	Alcohol use emergency plan	N/A
Session 9	Tackling craving	Feedback moment
Session 10	Restructuring thoughts	Feedback moment
Session 11	Rejecting alcohol offers	Feedback moment
Session 12	Evaluation of the Blend-A main module	N/A
Session 13	Selection of themes	Feedback moment
Session 14	Theme: Social skills—small talk	Feedback moment
Session 15	Theme: Social skills—tackling criticism	Feedback moment
Session 16	Theme: Social skills—giving criticism	Feedback moment
Session 17	Theme: Tackling feeling sad and moody	Feedback moment
Session 18	Theme: Tackling stress	Feedback moment
Session 19	Theme: Solving problems effectively	Feedback moment
Session 20	Theme: Tackling relapse	Feedback moment
Session 21	Introduction to maintenance	N/A
Maintenance month 1	Alcohol status and Quality of life	N/A
Maintenance month 2	Alcohol status and Quality of life—your assessment	N/A
Maintenance month 3	Alcohol status, Quality of life—your assessment, and Support	N/A
Maintenance month 4	Alcohol status, Quality of life—your assessment, and Support—your assessment	N/A
Maintenance month 5	Alcohol status, Quality of life—your assessment, Support—your assessment, And Motivation	N/A
Maintenance month 6	Alcohol status, Quality of life—your assessment, Support—your assessment, Motivation—your assessment, and Evaluation of the maintenance phase	N/A
Diary	Alcohol use	N/A
Diary	Evaluation of turning alcohol offers down	N/A

^a^Blend-A: Blending Internet Treatment into Conventional Face-to-Face Treatment for AUD.

^b^N/A: not applicable.

### Recruitment and Data Collection

To explore patients’ experiences with bCBT, we decided to conduct a qualitative study involving the 30 most recently enrolled patients in the Blend-A study who utilized the option of combining FtF sessions with internet-based sessions. We invited them to participate in either telephone-based interviews or FtF interviews to share their experiences. Of these 30 patients, 13 agreed to participate, while the others opted out or could not be reached.

Data were collected through semistructured individual interviews using an interview guide (Multimedia Appendix 1). The guide was developed based on themes identified as important in earlier studies [5,8,15-17]. The questions were open-ended, asking patients about their background, experiences with digital technology in their daily lives, motivations for choosing the offered internet-based treatment, and their experiences with the program during their AUD treatment. The guide was not pilot-tested. The interviews were conducted by the first author (KT), a female anthropologist and PhD, who was a postdoc at the time of the study with approximately 10 years of training in qualitative research. No prior relationship was established between KT and the patients before the interviews. As a result, the patients had no prior knowledge of KT and were informed that she was a researcher not connected to the treatment institutions. The interviews lasted between 9 and 43 minutes, with a mean duration of 30 minutes. No one was present at the interviews besides the patient and the researcher; no repeat interviews were conducted, and no field notes were taken. The interviews were audio-recorded, transcribed in full in NVivo (Lumivero), and checked for accuracy. Transcripts were not presented to the patients for feedback. Data saturation was discussed within the research group. All data were anonymized and securely stored.

### Data Analysis

The transcribed interviews with patients were qualitatively analyzed using the thematic analysis approach [18]. Thematic analysis is a method for identifying and describing patterns or themes within data, without being constrained by a preexisting theoretical framework. In this study, thematic analysis was used as a realist or essentialist method, focusing on reporting the experiences of the patients. Patterns and themes within the data were identified inductively and expressed in multiple ways. First, RC and KT read the transcripts multiple times independently and coded the interviews using headings that captured the essence of specific excerpts. Second, the headings and the content under each heading were discussed between the 2 coders. In cases of disagreement, either the heading was revised to better reflect the content or the content was moved to a different heading that more accurately defined it. Lastly, RC collated the categories with related content into overall themes using the qualitative software system NVivo. The themes were then refined by KT. Overall, the identified themes represented the participants’ first-person perspectives on bCBT, contributing new knowledge on aspects important to treatment. The final analysis was written with relevant quotations included, but no patients provided feedback on the findings.

### Ethics Considerations

The study was conducted according to current ethical standards. The protocol was approved by the Scientific Research Ethics Committee of the Region of Southern Denmark (project identification number S-20190166G). The Danish Data Protection Agency gave permission to collect and store data (record number 20/12692). After receiving oral and written information about the study, the patients signed a consent form.

## Results

### Sample Description and Thematic Analysis of Patient Experiences

A description of the sample of participants is presented in Table 2. The forthcoming analysis of the transcribed interviews is structured as follows: (1) patient experiences of how bCBT impacts treatment, comprising 4 themes; and (2) patient experiences with the practical use of the platform, also comprising 4 themes. An overview of the identified categories, themes, and subthemes is available for reference in Table 3 and will be described in detail in the following sections.

**Table 2:** Participant sample description (N=13).

Characteristics			Values^a^
**Sociodemographic**			
	Age (years)	53 (31-75)	
	Sex (female)	4 (31)	
	Married/in a relationship (yes)	9 (69)	
	Short, intermediate, or long education (yes)	13 (100)	
	Employment (yes)	7 (54)	
	On leave/early retired/retired (yes)	6 (46)	
**Alcohol use**			
	Excessive alcohol use (yes)	13 (100)	
	Using alcohol every day (yes)	5 (38)	
	Using alcohol for self-medication, for example, anxiety, depression, pain, sleeping (yes)	4 (31)	
	Had sought treatment to reduce or stop alcohol use (yes)	13 (100)	
**Technology use**			
	Familiar technology user (yes)	13 (100)	
	Feeling safe using the internet for treatment purposes (yes)	13 (100)	
	Had completed the platform sessions at the time of the interview (yes)	7 (54)	
	Had used the blended format (yes, as opposed to primarily using the platform)	10 (77)	

^a^Presented as mean (range) or N/n (%).

**Table 3:** Identified categories, themes, and subthemes (N=13).

Categories and themes			Frequencies for naming the themes, n (%)		Subthemes
**Patient experiences of how bCBT^a^ impacts treatment**					
	bCBT adds anonymity and privacy to the treatment course	6 (46)		bCBT helps when I find it transgressive to go to the clinic in person bCBT helps when I fear running into someone I know at the clinic	
	bCBT adds flexibility to the treatment course	8 (62)		bCBT fits well when my transportation options are limited bCBT fits well with my work life bCBT fits well with my home life	
	bCBT enables time and space for reflection	4 (31)		I can think about it at my own pace when I use the platform I have thought about it, when I attend a session, leaving room for new angles I will continue to think about it after the session, qualifying it	
	bCBT increases an assisted form of autonomy	N/A^b^		I have to take responsibility for my own rehabilitation process I want to go through the process on my own, with support from the therapist Constructive feedback leads to further progress in my change process The interaction between the 2 formats leaves room for deeper discussions	
**Patient experiences with the practical use of the platform**					
	User-friendliness	9 (69)		The internet-based treatment program is user-friendly, intuitive, and easy to use I appreciate how the program is tailored in accordance with the feedback given by the therapist	
	Written material and assignments	6 (46)		The online material is well-written and usable for acquiring knowledge and facts about drinking Reading the material helps me gain a greater reflection on my drinking habits The material can be a good starting point for a conversation about drinking	
	Diary and registrations	8 (62)		I used the diary for self-reflection, taking the time to think and evaluate my thoughts and feelings during the rehabilitation process It helped me to gain insights into my use of alcohol, habits leading to cravings, and how to deal with cravings This facilitated an internal dialog about whether the process was progressing as I hoped for	
	Motivating reminders	5 (38)		Reminders are helpful and well-intended There is a person behind the platform who expects my attendance at the program This adds to my motivation and progress in the treatment course	

^a^bCBT: blended cognitive behavioral therapy.

^b^N/A: not applicable.

### Patient Experiences of How bCBT Impacts Treatment

#### Key Themes in bCBT Patient Experiences

This category includes 4 themes: how the possibility of bCBT added anonymity, privacy, and flexibility to the treatment course; how bCBT provided time and space for reflection for the patients; and how bCBT fostered a supported form of autonomy for the patients. Each of the 4 themes is presented in detail below.

#### bCBT Adds Anonymity and Privacy to the Treatment Course

Some patients felt that the relatively private or anonymous nature of bCBT influenced their decision to choose this particular treatment format.

One factor that influenced the decision to engage in bCBT was its ability to reduce the uncomfortable experience of entering a clinic:

To me, it is very transgressive to sit in a waiting room in the clinic. So, I think it is nice that some of it is private. That is primarily why I wanted online

Patient, ID 1273

Another factor that made bCBT attractive was that it minimized the risk of encountering someone the patient knew at the clinic:

During this time where I have been attending the clinic, I have been offered group therapy and group meetings. And I have plainly refused it, terrified of meeting someone I know

Patient, ID 1268

For them, entering a treatment center required a significant amount of courage; staying at home and avoiding frequent visits were perceived as less anxiety-provoking.

#### bCBT Adds Flexibility to the Treatment Course

The majority of patients indicated that the flexible nature of bCBT motivated them to actively seek out or engage in the treatment. They found that the ability to participate from home made it easier to incorporate the treatment into their everyday lives. One important factor influencing this was limited transportation options, as illustrated in the following example:

You do not have to involve others in the fact that you are leaving for treatment. And I will say that, especially when you live in a thinner populated area as we do. [...] There are not so many options. And you must drive for everything. Just that you don’t need to drive for it. That makes a big difference

Patient, ID 1114

Consistent with the previous example, patients found bCBT easier to integrate into their work lives. They expressed that adding weekly FtF CBT to their already busy schedules would be difficult, if not impossible. The option to engage in bCBT alleviated these obstacles, as explained by one of the patients:

I was also at work and had to go in there and I did not want it to become too stressful for me. Then I think that it is fine and with the simultaneously sessions in person sometimes. I have been happy about this. [...] also, because I think it could stress me a little if I had to go in there all the time with work and the late hours [...] I think it was good because it took away the stress

Patient, ID 1256

Thus, the availability of bCBT offered patients a pressure-reducing model for receiving treatment, either partly or completely. They found bCBT convenient because it allowed them to engage with the treatment course at times that suited their home life. This was elaborated on by one of the patients:

And you can control it in your own tempo. That is also probably why I also wanted something online. [...] Every other week, I spend a lot of time with my partner. And it is suitable that the weeks where I am not then I can focus more on this.

Patient, ID 1273

The patients overall felt that bCBT provided a flexibility that aligned well with their daily lives. The ability to engage with the online format at times that suited each individual was viewed as a significant advantage to the treatment.

#### bCBT Enables Time and Space for Reflection

About one-third of the patients explained that they chose to receive bCBT because it was more likely to support them at their own pace in the rehabilitation process compared with FtF sessions alone. Working with the program at home allowed for a more thoughtful process, facilitating deeper realizations than those possible in a FtF CBT session with limited time. One patient elaborated on how using the platform at home provided uninterrupted time for reflection, leading to more comprehensive responses:

At home you are able go to and from. Understood in that way that you can answer more nuanced because you don’t have to do it right here and right now. But you can spend some time reflecting on the questions, your thoughts, and reaction patterns

Patient, ID 1184

The patients were committed to the idea of completing assignments at home in a peaceful and quiet environment, allowing them to reflect on their answers as they worked through the tasks. This reflection time and level of nuance made the treatment course feel more thorough—and even more effective—for them.

Second, another factor that made patients find bCBT more effective than they had initially imagined FtF CBT alone would be was the reflection time, which enriched their dialog with the therapist during FtF sessions. One patient explained:

I think having the background information and assignments to do in the program and think about and write down what it meant to you that is something that can make you think before you show up for the session and then you can unfold your thoughts at the session better than if you first were to start doing the thinking when you are in the room

Patient, ID 1350

The patients felt that this made the FtF sessions more fruitful, as the reflection time between completing assignments and meeting with the therapist allowed space for new thoughts and perspectives. They experienced that this reflection time made the FtF sessions more intense and effective, leading them to emphasize that it had the potential to enhance the effectiveness of FtF CBT.

A third and final factor influencing the patients’ perceptions of bCBT as being more thorough than solely FtF therapy was that the reflections continued even after the sessions. This was explained by one patient:

It had two functions. One was the immediate when I was working with the assignments where I was thinking quite some about the things in the assignment. And then, the other thing was, in the days after it kept being there in my head and I could continue working and think and use some of the things that I had been working with

Patient, ID 1316

The topics discussed on the platform and by the therapist during FtF CBT remained part of the patients’ reflections throughout their rehabilitation process. In this way, bCBT facilitated increased self-reflection during the treatment course—before, during, and after the FtF sessions.

#### bCBT Increases an Assisted Form of Autonomy

The patients felt that the combination of platform use and FtF sessions enhanced their treatment experience. One factor influencing this was the realization that they needed to take their alcohol-related problems seriously and assume responsibility for their own rehabilitation. This was explained by one patient:

First and foremost, it has been an important factor in making me more conscious about the fact that I had a huge problem with alcohol that I had to do something about. And for the first time in my life, I was deeply aware about that it was the most important thing I could do for myself. And it has contributed into making that clear for me somehow. Because the themes and the subjects that were raised were relevant and it was done in a proper manner that I could understand and relate to

Patient, ID 1284

The patients further explained that personal motivation is essential for recognizing the need for change. Another important aspect closely connected to their sense of autonomy was the opportunity to engage with the program in private. One patient elaborated:

I wanted it to myself. It was moments that I wanted to myself. And I did not want to explain why I went down here. Or I could but I did not need all the questions. I wanted it to myself together with the therapists. It was my little world

Patient, ID 1345

The patients distinguished between the rational decision to seek treatment and the deeper, personal realization of needing to make a significant change. For them, it was much more than simply concluding that entering treatment was the healthiest or most appropriate choice. Once they decided to pursue treatment, they emphasized the importance of taking control over their lives without external interference—an autonomy that bCBT fosters and strengthens.

The autonomy offered by bCBT was, however, a supported form, endorsed by the therapist. Patients described receiving constructive online feedback from their therapist as a key element in advancing their change process. They found the feedback to be an acknowledgment of their efforts, often including reflections on their completed assignments, along with additional suggestions for new, motivating challenges. One patient elaborated:

It had two functions. One was to point at things that I should be attentive to or work more with. And at the same time, it also gave me the feeling that someone else than me could see that there was a meaningful process in what I was doing and that was fine

Patient, ID 1316

A final note on how the combination of the 2 intervention formats assisted the rehabilitation process, as experienced by the patients, was that completing some sessions on the platform freed up time during FtF sessions to focus on deeper issues that required the therapist’s in-person perspective. One patient elaborated:

After I started using the app there were some things that the therapist did not have to spend time explaining to me, which they otherwise would have done in the sessions. But when you only have one hour every other week then it is nice that you don’t have to spend so much time explaining. Then I think it is nice that you can just dive into how it works for you, personally

Patient, ID 1350

The patients found that being able to read background information before the FtF CBT session with the therapist saved time for deeper conversations during the session. As a result, they felt that the combination of completing assignments at home and attending FtF CBT supported them more than they had anticipated from solely participating in FtF sessions.

### Patient Experiences With the Practical Use of the Platform

#### Patient Engagement and Usability in Treatment: Key Themes

This category includes 4 themes that describe user-friendliness and patient engagement with written materials, assignments, diaries, registrations, and motivating reminders. Each of the 4 themes is presented in detail below.

#### User-Friendliness

Almost all patients found the internet-based treatment program to be user-friendly. Upon introduction to the program, they found it manageable, intuitive, and easy to use. They appreciated how the program was tailored based on the feedback provided by the therapist. One patient explained the usability as follows:

I answered the assignments and sent them to the therapist for feedback on the scheduled day for her to give that. That means I followed the rhythm in the program and that worked well.

Patient, ID 1316

The setup of the reading material and assignments were important, as was the digital component, the patient added:

It has worked very well for me because it is easy access, and I can do it regardless time and space. I would also be able to do that with a piece of paper but that would be heavier. It would certainly be a much heavier process to work with it on paper. It has also been easy for me sometimes to go back in the process and see what I was thinking at that time. What was it that I was talking to myself about at that time. And then I could go forward again. Very simple

Patient, ID 1316

Having the ability to navigate back and forth between completed assignments and new ones was found to be helpful in initiating their rehabilitation process.

Visual aids were also mentioned; 1 patient appreciated the animated films for providing an informal yet understandable overview of the problem area. Another found the videos too polished, while a different patient found the tools presented in the videos to be very helpful. When encountering technical challenges, one suggested asking the therapist for assistance to improve navigation within the program. We also inquired whether the patients had missed anything on the platform or had suggestions for adjustments for future iterations. Their needs and wishes for the platform can be seen in Textbox 1.

**Needs and wishes for the further development of the platform.**

- Tempering expectations may be helpful before starting an online treatment course.
- The examples in the assignments should be more diverse, addressing a wider range of patient groups.
- Include a videoconferencing option in the program, allowing patients to choose video sessions with the therapist instead of FtF sessions.
- It should be more intuitive to navigate the treatment program and easily go back and forth to find relevant sections to read, watch, or fill out again.
- Being able to reply directly to the therapist’s feedback, rather than having to copy it into a separate message, would be helpful.
- Having the written material reviewed by a communication or rhetorical specialist who is not otherwise connected to the field would be beneficial.
- It would have been more relevant for some if the program better reflected that patients could also be in control, highlighting the potential for a process stemming from that.
- A module focused on pain relief.
- Free navigation and log-in within the program.
- More challenging assignments, examples, and questions would have enhanced the program’s relevance.
- A timeline showing your progress in the program would provide a better overview.
- An individualized program where different answers lead patients to tailored treatment paths based on their unique problems and needs.
- Would have preferred the entire program, not just the diary and registrations, to be smartphone app-based.
- Would have liked the option to access additional information and assignments beyond the released session, as there could be a significant wait for feedback and the next session.
- A function that allows you to upload pictures of written materials.
- A clearer presentation of the self-selected themes.
- That the program involved families, provided explanations about alcohol use, and included monitoring of their observations while addressing the challenges they might encounter.
- The program could do more to celebrate victories and motivate users by incorporating visual positive reinforcements.

#### Written Material and Assignments

Overall, the patients found that the online material was well-written and effective for acquiring knowledge about how their drinking problem affected them. Reading the material helped them gain greater insights into their drinking habits, providing information they did not have beforehand. They also noted that the written material was occasionally patronizing in its choice of wording, emphasizing that this could potentially have a negative effect on patients. One final observation was that the reading material could serve as a good starting point for conversations with those close to the individual struggling with alcohol use.

The patients found that the assignments and tasks were relevant and aligned with their own experiences regarding problematic drinking. However, one patient felt that some tasks were somewhat superficial and requested a more challenging and pertinent program. Many patients noted that if the assignments lacked meaningful content related to their everyday lives and specific challenges, they found it difficult to answer the questions. They emphasized the importance of including a broad range of questions and examples in the assignments. One patient elaborated on this point:

It is difficult to identify with the questions and then the answers also become irrelevant. And I think that the learning that was intended with questions and answers it does not appear

Patient, ID 1273

The patients emphasized that it can be challenging to formulate questions that resonate with every individual. To overcome this obstacle, some patients suggested a practical solution: the program could offer options for patients to select assignments that are relevant to them while allowing them to skip those that are not. One patient elaborated:

Because it is super complex why you do what you do, which factors that is a starting point for a misuse. And I don’t think that that complexity can be reflected in such a very linear progress. I’m sure that there are good considerations behind, but I think that if it needs to be relevant for both the well-functioning drinker and the one who are not in control at all, I think you need to differentiate it

Patient, ID 1273

The patient highlighted the complexity inherent in being an individual with a drinking problem, noting that this makes it challenging to organize assignments in a way that aligns with each person’s unique experiences.

#### Diary and Registrations

The majority of patients reported using the diary and registration function on the platform. However, a few felt that the diary primarily concentrated on misuse and relapse, along with follow-up questions. For them, this focus was not relevant, as they aimed to lower their consumption; they would have preferred the program to also highlight strategies for reducing drinking.

The remaining patients found it rewarding to gain insights into their own alcohol use, the habits that led to cravings, and strategies for managing those cravings. They noted that using the program encouraged self-reflection, allowing them to take the time to think about and evaluate their thoughts and feelings throughout the process. The patients emphasized that this self-reflection facilitated an internal dialog about whether their rehabilitation journey was progressing as they hoped. As one patient expressed, regarding the use of the diary and registration features:

I had good help from my therapist to become aware of what happened inside of me. At one point, I just wrote number of units and clicked ‘send’. And then she asked me why I did not fill out the rest with feelings, thoughts, and where I was etc. And then I said, it is because it is the same every day and it is just a routine now. And then she convinced me to try. She said, something still happens in your thoughts and in your feelings and in your body, try to really feel. And it was quite interesting because it was maybe also especially through that that I realized that this is about being afraid of choice. One big avoidance maneuver from my side against meeting my thoughts when I go to sleep. Corresponding, in the periods where I managed not to drink, then she asked me to also register what happened emotionally, mentally, and bodily. And it was quite good exercises, especially with her guidance, where I managed to dive into it because it is so easy for me to scroll over it

Patient, ID 1268

The patient expressed how the therapist guided and motivated them to engage more deeply with the program to fully understand the nuances of alcohol misuse. This written reflection enhanced the patient’s awareness of their thoughts, making previously vague issues surrounding their alcohol use more tangible. As a result, this clarity contributed to progress in reducing their alcohol intake or achieving abstinence. Most patients noted that during the FtF CBT sessions, the therapist would begin the discussion where the written feedback had concluded, enriching the conversation with additional perspectives. This approach made the dialog more nuanced than the written feedback alone. One patient described how the concise nature of their written assignment answers and the subsequent feedback was designed to be expanded upon during the conversation.

I imagine that if the therapist had produced more comprehensive written feedback, one could be nervous that the question or conversation would close with the answer he then gives. And then it doesn’t have the conversation-generating effect when you show up for the next session because you can be of the understanding that the subject is closed. And typically, it is not a subject that can or should be closed but ought to be unfolded during more future conversations

Patient, ID 1350

Most patients found it helpful to receive brief written feedback on their completed assignments, which they could then actively discuss during the FtF CBT sessions. However, a couple of patients expressed concern that the work they had done in the online treatment program did not always connect with the topics addressed by their therapist during the FtF sessions.

#### Motivating Reminders

About one-half of the patients felt motivated by receiving reminders from their therapists when they had not engaged with the program for several days. One patient elaborated on this point:

I also think that the therapist in the other end was good at following up on my platform use. If I was too slow, she wrote to me. Or not if I was too slow, but she made sure to follow up so the whole thing did not come to a standstill, I think. [...] And it worked very well. The motivation was rising. And when you get a reminder like that, you know there is another person in the other end, and then you also become motivated to do something about it, I think

Patient, ID 1320

The patients felt that knowing there was a person behind the platform expecting their engagement added to their motivation and progress in the treatment. Although the reminders might come across as somewhat supervisory, they were viewed as helpful and well-intended.

## Discussion

### Overview of Findings

In this study, we found that users experienced several advantages of using iCBT, including increased anonymity and privacy. Most importantly, it offered flexibility in the treatment process, allowing patients to focus on their rehabilitation at their own pace. The availability of written material in the online program was appreciated, as it provided valuable knowledge about AUD and its effects on individuals. Systematic access to written material that could be revisited helped patients better understand the mechanisms behind their problematic drinking—knowledge they felt they had not previously received. For some patients, the written content also served as a useful starting point for discussions with relatives, reducing the time therapists needed to spend on explaining background information and facts about AUD. We noted that patients found the platform particularly beneficial for gaining insights into their alcohol problems compared with solely FtF treatment. The assignments not only facilitated acknowledgment of their issues with alcohol but also encouraged self-reflection before FtF sessions, allowing for deeper exploration with the therapist. Reminders were well-received and helped motivate patients to complete assignments. Only a few disadvantages of combining internet-based iCBT with FtF treatment were mentioned.

Consistent with previous studies, patients in this study found the iCBT program user-friendly, describing it as intuitive and easy to navigate. However, a small number of patients reported difficulty answering some questions within the program, as they felt those questions were not relevant to their specific issues. They suggested that the assignments should include more examples to better address a wider range of patients and to make the program more personalized. This aligns with findings from various studies indicating that online programs often fail to meet all patients’ individual needs, leading to frustration [7] and decreased motivation [7,17,19]. Reduced motivation can also stem from programs being overly time-consuming or demanding [7,20].

Our findings also support those of the qualitative study by Khadjesari et al [8], which examined people searching for online help to reduce their drinking, as well as the user experience study on internet-based treatment for problematic alcohol use by Ekström and Johansson [5]. Both studies concluded that some individuals felt embarrassed about their AUD and preferred to keep their struggles private. Ekström and Johansson [5] also reported that some patients feared that if local authorities became aware of their drinking problem, they might risk losing their children or jobs, which led them to avoid attending alcohol treatment centers. We found that a key aspect of choosing a blended treatment course was the added anonymity and privacy it provided for patients. Practical factors that influenced their decision to select the online format included the flexibility it offered, making it easier and more convenient to integrate into their daily lives, including family commitments, work, and transportation challenges.

Perhaps more importantly, we found that the time patients spent online on tasks they could complete independently seemed to free up time for FtF sessions with the therapist, allowing for deeper therapeutic exploration of more complex issues. This phenomenon is known as the “flipped clinic” [21,22], where digital resources are used for aspects of treatment that do not require the therapist’s presence, thereby reserving in-person time for when it is most needed. This finding aligns with the qualitative study by Black et al [1] on user motivations and experiences with an internet intervention for alcohol reduction, as well as with Hadjistavropoulos et al [16], who explored patient perspectives on the strengths and challenges of therapist-assisted iCBT. Both studies found that patients appreciated the flexibility of online treatment, allowing them to complete the course at their own pace and use it as a substitute for FtF CBT when necessary. This conclusion was also reached in the review by Verhoeks et al [7], which discussed women’s expectations and experiences regarding eHealth treatment. It suggests that eHealth may be seen as a complementary or integrated aspect of traditional health care rather than as a standalone solution. Therefore, iCBT could serve as an important tool in the recovery process to help prevent relapse. Patients who are already familiar with the treatment content may find it easier to engage with an online program when needed, or conversely, they might feel more comfortable interacting with a therapist after gaining foundational knowledge from online modules. Further studies on the effects of flipped clinics are warranted.

Most patients in this study found that using a digital diary enabled them to track changes in their alcohol consumption, recognize triggers for cravings, and practice strategies for managing those cravings, ultimately strengthening their sense of autonomy. Furthermore, as noted by Ekström and Johansson [5] in their user experience study, patients expressed appreciation for the therapists behind the screen, praising their professionalism and support. The therapists were perceived as honest and genuine in their approach, which enhanced the patient’s experience by providing helpful feedback and supporting their change process. This finding is consistent with the results of this study.

Patients expressed that the desire for change regarding their alcohol problem should originate from within themselves. This introspection required time for self-reflection at their own pace, which they felt was facilitated by bCBT. This aligns with findings from the user experience study by Ekström and Johansson [5], where access to written text led to a high level of recognition among patients, who remarked, “that’s why I react like I do or behave as I do.” In a patient perspective study on iCBT for alcohol misuse, Hadjistavropoulos et al [17] similarly found that written material from iCBT enhanced self-awareness and provided insights into patients’ problems with AUD, helping them identify reasons for making changes. In a related qualitative study on experiences of iCBT for depression and anxiety, Lindegaard et al [6] also reported that patients found the written texts informative. These texts were perceived as helpful in changing routines, limiting negative thoughts, and fostering a shift toward more positive thinking. In summary, we aim to highlight that self-reflection is essential for patients with AUD to strengthen their autonomy. This autonomy is further supported when FtF CBT is combined with iCBT in a blended treatment approach (bCBT).

Increased autonomy through self-reflection involves a deep and conscious examination of thoughts, as well as recognizing the origins and causes of one’s feelings, emotions, and actions. Achieving this level of reflection requires a substantial amount of uninterrupted time, which is often challenging in FtF CBT sessions where time is limited. In this context, bCBT facilitates an effective interaction by providing a valuable combination of time, quietness, and contemplation for the patient. The patients in the Blend-A study emphasized that using the platform before FtF sessions allowed the therapist to start the session based on the patient’s feedback from the assignments. This approach fostered a more engaging, in-depth, and meaningful conversation between the therapist and the patient. Therefore, the patients found that both the iCBT and the FtF CBT sessions with the therapist contributed significantly to their reflection process. This dual approach enhanced their sense of perceived autonomy, which in turn improved their ability to succeed in treatment and ultimately in their rehabilitation.

In general, autonomy is often understood as *substantive independence* and *self-determination* [23,24]. However, in this context, the concept of relational autonomy [25,26] appears more relevant. Relational autonomy emphasizes the influence of situations marked by oppression or deprivation—such as struggles with alcohol—and recognizes how external factors can constrain an individual’s autonomy and ability to act. It also considers how values, attitudes, and standards associated with these constraints can shape an individual’s perception of what they believe they can achieve and how they can perform [27]. Based on studies of traumatized individuals, Bernstein [28] suggested that people may confront “underlying and intractable dimensions of vulnerability, dependence, and potential helplessness that are normally hidden from consciousness.” We propose that the journey of transitioning from alcohol addiction similarly involves an experience of relative vulnerability, dependence, and helplessness during the change process. Before deciding to seek treatment, patients with AUD may have experienced a loss of self-care, increased cravings that lead to diminished control, and a lack of awareness of their surroundings—conditions that self-reflection can painfully illuminate. In the Blend-A study, we found that patients recognized how engaging with iCBT assignments heightened their awareness and sensitivity to their drinking behavior, enabling them to reflect on their issues in a broader and deeper context than what they could achieve through FtF sessions alone. This finding is supported by a qualitative study in which therapists noted that patients were more likely to attribute their treatment success to themselves in the context of online therapy compared with FtF treatment [29]. In our study, therapists facilitated the patients’ autonomy by bridging the gap between the forward-looking goal of achieving abstinence or reducing alcohol intake and the self-realization process that took place in a private setting.

Moreover, when viewed through the lens of relational autonomy, the patients in this study found that the diary function helped them better understand themselves, recognize what was happening in their lives, and assess their progress throughout treatment, thereby strengthening their autonomy. Some patients reported that reminders from the therapists were motivating, especially if they had not engaged with the program for several days. This finding aligns with previous studies that concluded check-ins serve as motivating factors, helping patients hold themselves accountable [30]. Some patients described the feedback from their therapist as positive, acknowledging, and supportive, viewing it as a solid foundation for subsequent FtF CBT sessions. They appreciated that the therapists tailored their feedback to benefit each patient, aligning the feedback objectives with the outcomes of the patient’s self-reflection. For example, the ability of a patient to drink less may not initially hold intrinsic value in isolation. However, if through self-reflection the patient recognizes the need to stop drinking for reasons such as health, family, or job security, then that realization aligns the treatment goals with a meaningful emotional experience. The therapist then helps the patient develop an action plan to achieve the goal of reducing or eliminating alcohol consumption. For this endeavor to succeed, both the patient and the therapist must actively engage with each other, identifying new opportunities and goals through collaboration.

In summary, successful cooperation may not always be achievable if the patient is not genuinely committed to the treatment, particularly in settings with limited time and insufficient opportunities for careful reflection. This highlights the importance of therapists helping patients cultivate a balanced sense of autonomy, while also addressing the risks of losing control over alcohol, experiencing relapse, or discontinuing treatment. Patients especially noted that they used the online program to read materials and reflect on assignments before their clinic visits. This approach may have allowed them to advance further in their treatment than they might have with FtF sessions alone.

### Limitations and Strengths

This study has both limitations and strengths. One limitation is that the interview guide was not pilot-tested, which may have influenced how themes were addressed and how questions were posed, potentially impacting participants’ responses. Additionally, the lack of stakeholder checks on the transcripts and codes means there was no external validation of the findings [31]. As we approached the 30 most recently enrolled patients in the Blend-A study, selection bias may have occurred during recruitment. This could have resulted in a less representative participant sample, potentially affecting the external validity of the results [32]. Moreover, it may introduce bias into the findings that all patients considered themselves skilled technology users and felt comfortable using the internet for treatment purposes. However, this aligns with the distribution of digital technology use in the general Danish population, where 97 out of 100 families had internet access, 96% owned a mobile phone, and 91% had a computer in 2020 [33].

We consider it a strength that the same researcher interviewed 13 patients for this study, as this number is, according to Guest et al [34], sufficient to achieve data saturation, allowing for an inductive analysis process where significant themes can emerge from the raw data. This number of participants also facilitates a stronger interviewer-interviewee alliance, which enhances the validity of the interviews [35]. Additionally, the credibility of the study findings is bolstered by the fact that 2 researchers conducted the coding of the raw data to ensure internal validation [31,36,37].

### Perspectives and Implications to Practice

In this study, we found it essential to consider all the reasons for introducing new technology, ensuring that users understand the exact nature of the application. For users, technology cannot stand alone; ideally, it should not replace FtF treatment. Instead, it should be clear to users how technology can serve as an additional resource in treatment and potentially enhance the overall quality of care. If this is not taken into account, there is a risk of diminishing users’ desire and enthusiasm for new treatment approaches that incorporate technology.

### Conclusions

In conclusion, our findings indicate that patients engaging in bCBT perceive more benefits than drawbacks associated with this blended intervention. bCBT enhances anonymity, privacy, and flexibility, providing ample time and space for reflection. Essentially, it offers patients a form of assisted autonomy that cannot be achieved through iCBT or FtF CBT alone. The combination of these approaches allows patients to fully appreciate the benefits of treatment, as they have the opportunity for self-reflection, supported by their therapist between FtF sessions.

## Appendix

Multimedia Appendix 1 Interview guide.

## Abbreviations

AUD: alcohol use disorder
bCBT: blended cognitive behavioral therapy
Blend-A: Blending Internet Treatment into Conventional Face-to-Face Treatment for AUD
CBT: cognitive behavioral therapy
COREQ: Consolidated Criteria for Reporting Qualitative Research
FtF: face-to-face
iCBT: internet-based cognitive behavioral therapy
