# Supplementary material for: Patient Perspectives on Blended Internet-Based and Face-to-Face Cognitive Behavioral Therapy for Alcohol Use Disorder: Qualitative Study
Source: J Med Internet Res. 2024 Oct 23;26:e47083. doi: 10.2196/47083 (PMC11541155; doi:10.2196/47083)
Supplement: Multimedia Appendix 1 [file jmir_v26i1e47083_app1.pdf]

**Appendix 1: Interview guide for patients who have been using blended internet-based treatment for alcohol use disorder.**	
**Introductory questions:**	*Thank you for participating in this interview. If you consent, I would like to record it. The interview will be about how you have experienced combining conversations by physical presence with internet-based alcohol treatment. First, I will pose some introductory questions, and then some questions about your reasons to choose internet-based treatment, and some questions about how it has been to use it. I will finish of with some concluding questions. I will start asking questions, you are welcome to explain and describe in the direction the experiences and perceptions occur to you. At first, the introductory questions.*<br>• Do you live together with someone? If yes, who?<br>• Do you have an education and occupation? If yes, what?<br>• Will you describe a normal every day for you?<br>• Do you use a computer/iPad in your everyday life (except for internet treatment)? If yes, how often?<br>• Do you feel safe using the internet under treatment?<br>• Do you suffer from any diseases?<br>• What were your reasons for choosing alcohol treatment (Do they explain how long, how much and how often they have been drinking – and their motivation searching for treatment)?
**Questions about choosing combined treatment course:**	*Is there anything else you would like to tell about your background? Otherwise, I will move to the questions about your reasons for choosing a combination of conversations by physical presence and internet-based alcohol treatment as your care trajectory.*<br>• What considerations did you have when you had to decide whether you would like only treatment by physical presence or you would like to combine the course with internet-based treatment?<br>• How have you experienced having the opportunity to choose between physical presence and the combination with interned-based treatment?<br>• Would you want to start treatment if there was no opportunity for the internet-based/combined course?
**Questions about experiences with using the program:**	*Is there anything else you would like to tell about reasons for choosing the combined care trajectory? Otherwise, I will move to the questions about your experiences with using internet-based treatment. First, some questions about the program specific.*<br>• How have you experienced using the internet-based treatment program?<br>• What is your impression of the material in the program (reading material, tasks, diary, and registrations)?<br>• How has it been to execute the tasks in the program?

	• How have you experienced getting feedback in writing from your therapist?<br>• Have you experienced any challenges when using the program? If yes, which?<br>• Do you have any ideas as to how the program can improve?
**Questions about receiving combined/internet-based treatment:**	*Then some questions about receiving the combined/internet-based treatment in general.*<br>• Does the internet-based treatment support your everyday life? How?<br>• Have you experienced the combination with internet-based treatment can do something the physical presence alone can´t? If yes, what and how?<br>• Have you experienced any challenges because your treatment was combined or internet-based? If yes, which?<br>• Is there anything you have been missing in your care trajectory?
**Concluding questions:**	*Is there anything else you would like to say about your experiences with using the program? Otherwise, I will ask:*<br>• Is there more you would like to tell?<br>• Do you have any questions you would like to ask?<br>*Thank you a lot for participating in this interview and share your experiences. The interview will be anonymized and transcribed. Then the interviews will be analyzed and disseminated in an article.*
